# Horizontal Transfer of miR-643 from Cisplatin-Resistant Cells Confers Chemoresistance to Recipient Drug-Sensitive Cells by Targeting APOL6

**DOI:** 10.3390/cells10061341

**Published:** 2021-05-28

**Authors:** Grace R. Raji, Aswini Poyyakkara, Anjali Kunhi Krishnan, Ashutosh Kumar Maurya, Udeshna Changmai, Sharath S. Shankar, V. B. Sameer Kumar

**Affiliations:** 1 Department of Biochemistry and Molecular Biology, Central University of Kerala Periye, Kerala 671316, India; gracerajir@cukerala.ac.in (G.R.R.); aswinip@cukerala.ac.in (A.P.); anjali316@gmail.com (A.K.K.); ashu@cukerala.ac.in (A.K.M.); udeshnac7@gmail.com (U.C.); sharathshankar82@gmail.com (S.S.S.); 2 Department of Medicine, Thomas Jefferson University, Jefferson Alumni Hall, 1020 Locust Street, Philadelphia, PA 19107, USA

**Keywords:** cisplatin resistance, exosomes, miR-643, APOL6, apoptosis

## Abstract

Acquisition of resistance to cisplatin is a major impediment to the success of cisplatin-based combination therapies for cancer. Recent studies indicate that exosomal miRNAs derived from drug-resistant tumour cells can confer resistance properties to recipient cells by a horizontal transfer mechanism. Although the role of horizontal transfer of a few miRNAs has been described, little is known about the concerted action of horizontal transfer of miRNAs in conferring cisplatin resistance. The present study was designed to identify the role of miR-643, which is one of the most significantly increased miRNA in exosomes released from cisplatin-resistant Heptocarcinoma cells, in altering the cisplatin resistance properties of recipient cells. Drug-sensitivity assays involving miR-643 revealed that ectopic expression of miR-643 can desensitise the cells towards cisplatin. Furthermore, we identified APOL6 as a major target of miR-643. Further mechanistic studies showed that miR-643 can modulate APOL6 mRNA and protein levels, leading to a reversal of APOL6-mediated apoptosis. Altogether, our results suggest an APOL6-dependent mechanism for miR-643 mediated cisplatin resistance upon the horizontal transfer across cell types.

## 1. Introduction

Chemotherapy is one of the most commonly employed treatment methods for cancer management, which involves the use of cytotoxic agents [1]. The major hurdle in the success of chemotherapy is the development of drug resistance, where the cancer cell acquires the ability to evade the effects of cytotoxic drugs by manipulating their genomes and drug metabolism [2]. Drug resistance is a multifactorial phenomenon that can arise either due to factors that include altered drug efflux pumps (P-gp) [3,4], decreased drug activation [5], increased drug degradation and inactivation, enhanced DNA repair mechanisms [6], or blocking of programmed cell death [7]. Recent reports suggest that drug resistance is not only regulated by genetic or epigenetic changes, but also by a class of regulatory RNA molecules known as microRNAs (miRNAs) [8]. MicroRNAs are a family of 21–25 nucleotide small non-coding RNA molecules that regulates gene expression by interacting with the 3′UTR of target mRNAs to induce mRNA degradation or translational repression [9], thereby regulating a vast variety of biological processes such as embryogenesis, cell growth, cellular migration, invasion, and apoptosis [10,11,12,13]. Cisplatin is one of the most commonly used chemotherapeutic agents to treat many types of cancers [14]. However, prolonged use of cisplatin-based chemotherapy often leads to the development of cisplatin resistance [15]. Cellular resistance to cisplatin occurs as a result of (a) reduced intracellular accumulation of cisplatin due to defect in copper transporter 1 (CTR1) and (b) increased efflux of cisplatin by overexpressing ABC ATPases [16]. MRP2 is the major ATPase responsible for increased efflux of cisplatin in resistant cells [17,18]. CTR1 was reported to be downregulated in cisplatin-resistant cancer cell lines [19]. Although diverse molecular and cellular mechanisms have been suggested in the acquisition of cisplatin resistance, the exact molecular mechanism of cisplatin resistance and its regulations are poorly understood. Dysregulation of apoptosis has been reported as a key contributor to cisplatin resistance [20]. Cisplatin has been reported to induce mitochondrial SMAC and cytochrome c, which are two main factors involved in the mitochondrial death pathway [21]. Acquired resistance to cisplatin in non-small-cell lung carcinoma (NSCLC) cells has been reported to occur as a result of alteration in signalling, leading to reduced G2/M cell cycle arrest and apoptosis [22]. Multiple studies including ours have identified miRNAs as a key determinant of cisplatin sensitivity or resistance, since they can regulate a large network of genes involved in different signalling pathways and apoptosis associated with cisplatin resistance [23,24]. Since the evasion of drug-induced apoptosis is an important property of resistant cells, miRNAs can influence drug sensitivity by regulating apoptotic or antiapoptotic genes [25]. miR-15 and miR-16 have been reported to induce apoptosis by targeting Bcl-2 [26], whereas miR-21 functions as an anti-apoptotic factor [27]. In addition, the miR-17 cluster was reported to act either as a tumour suppressor or as an oncogene by regulating apoptotic or anti-apoptotic genes depending on the cellular context [28]. Mounting evidence suggests the role of cancer-secreted exosomes in conferring drug resistance. miRNAs carried by these exosomes could be transferred to recipient cells to exert an alteration of gene expression [29]. Recent studies including ours [24] have reported that cancer cell-secreted exosomal miRNAs mediate intercellular communication and can transfer MDR-associated miRNAs to target cells, thereby altering the chemosensitivity properties of recipient cells [30]. It has been reported that exosomal miR-1246 derived from MDA-MB-231cells can promote chemoresistance in recipient cells [31]. Exosomal transfer of miR-21 from stromal cells also has been reported to enhance paclitaxel resistance in recipient ovarian cancer cells by targeting APAF1 [32]. However, detailed studies on the exosomal miRNA expression pattern responsible for chemo-resistance is still lacking. In view of these facts, we tried to check the expression pattern of some candidate exosomal miRNAs from cisplatin-resistant HepG2 cells, which are not reported yet in drug resistance and functionally validate the role, if any of most significantly dysregulated of such miRNAs. The candidate miRNAs analysed include miR-554, miR-1258, miR-643, miR-1307, and miR-3194.

In view of the observations that micro RNAs packaged in the form of exosomes can alter the drug-sensitivity properties of recipient cells, and data from our previous published work that showed exosomes from cisplatin-resistant (Cp-r) HepG2 cells can enhance the cisplatin resistance properties of Hela cells towards cisplatin, this work is aimed to identify the role of exosomal miR-643, which is one of the most significantly increased miRNA, released from Cp-r Hepatocarcinoma (HepG2) cells in altering the resistance properties of recipient Hela cells towards cisplatin. miR-643 has been reported as a tumour suppressor in osteosarcoma cells, which can target ZEB1 and reduce tumour progression [33]. However, the role of miR-643 in drug resistance has not been reported yet, and we have explored that aspect in this study.

## 2. Materials and Methods

### 2.1. Cell Culture

Three cell lines were used, two parental (HepG2 and Hela) and one cisplatin-resistant subline. HepG2 (Hepatocellular carcinoma cell line) and Hela (Cervical cancer cell line) were procured from NCCS Pune and cultured in DMEM (Himedia, India) supplemented with 10% FBS, antibiotic antimycotic solution, and L-glutamine (Himedia, India). The cells were maintained under standard culture conditions of humidified atmosphere with 95% air and 5% CO_2_ at 37 °C. Anti-APOL6 antibody was obtained from Origin labs, Kerala. Anti beta actin antibody and cisplatin were purchased from Sigma Aldrich, St. Louis, MO, USA. Cp-r HepG2 cells were developed by exposure to increasing drug concentration. HepG2 cells were initially exposed to an IC_50_ dose of cisplatin, which was followed by gradually increasing the dose until the concentration of cisplatin was 3 times the IC50 value. The cisplatin-resistant (Cp-r) cells used in this study were 15-fold more resistant to cisplatin than cisplatin-sensitive (Cp-s) parental cells.

### 2.2. XTT Viability Assay

XTT assay was performed as described earlier [34]. Cells were seeded in 96-well plates at a seeding density of 3000–4000 cells/well and allowed to attach overnight. After attachment, the cells were treated with cisplatin (0.75–100 μM) and incubated further for 24 h. After incubation, the cells were treated with 25 μL XTT reagent (Invitrogen, Carlsbad, CA, USA) followed by incubation at 37 °C for 3–4 h. Then, absorbance was measured at 450 and 630 nm using Perkin Elmer multimode plate reader. From the OD values obtained, percentage cell viability was calculated, and IC_50_ values were determined by regression curve analysis.

### 2.3. Isolation of Exosomes from Culture Media

Exosomes were isolated from culture media using total exosome isolation reagent (Invitrogen) as per the manufacturer’s instructions. Briefly, cell culture media were collected and centrifuged at 200× *g* for 30 min to remove the cell debris. Thus, the supernatant obtained was transferred to a fresh tube into which a half-volume equivalent of total exosome isolation reagent was added, mixed, and incubated 4 °C at overnight. After incubation, samples were centrifuged at 10,000× *g* for 1 h at 4 °C. The supernatant was removed, and then, pelleted exosomes were resuspended in 1× PBS, which was followed by protein estimation and downstream experiments.

### 2.4. Isolation of Exosomal RNA and Real-Time PCR Analysis

Isolation of exosomal RNAs were carried out using an Exosomal RNA isolation kit (Invitrogen). The purity of isolated miRNA was checked using Agilent bioanalyser 2000 followed by cDNA synthesis and qRT-PCR analysis using SYBR green (Qiagen, Hilden, Germany) by Kang method [35].

### 2.5. Kyoto Encyclopedia of Genes and Genomes (KEGG) Pathway and Gene Ontology (GO) Analysis

Kyoto Encyclopedia of Genes and Genomes (KEGG) pathway enrichment analysis and Gene Ontology (GO) analysis were performed using the web-based tool miRNet (www.mirnet.ca (accessed on 24 October 2018)), and the threshold of significance was defined by a *p*-value < 0.05.

### 2.6. Prediction of miR-643 Target Gene and 3′UTR Analysis

The genes targeted by miR-643 were determined using a web-based tool miRNet and five online computational algorithms, viz., TargetScan, microrna. Org, Pictar, Diana and Target miner. 3′UTR of the predicted target was identified using a web-based tool TargetScan (www.targetscan.org (accessed on 10 December 2018)).

### 2.7. mRNA Stability Assay

Hela cells were seeded at a density of 2 × 10^5^ cells in 6-well plates. The cells after reaching a confluency of 70% were transfected with miRNA overexpressing plasmid (miR-643) along with proper empty vector control (pCMVmiR obtained from OriGene) using polyethyleneimine method. Then, 24 h post transfection, cells were treated with actinomycin (6 µg) to inhibit transcription for different time intervals such as, 0, 1, 3, 6, and 12 h followed by RNA extraction and Real-time PCR analysis of the mRNA target.

### 2.8. Luciferase Reporter Assay

The wild and mutant APOL6 3′UTR was cloned into a pMIRTARGET reporter vector downstream of luciferase gene. The mutant-type APOL6 3′UTR was made by deleting a miR-643 binding site. For luciferase reporter assay, HeLa cells seeded at a density of 2 × 10^5^ cells per well in 6-well plates, after reaching a confluency of 70%, were co-transfected with empty pmiR reporter plasmid or with pmiR vector containing wild-type and mutant APOL6 3′UTR in conjunction with miR-643 mimic or vector controls using Lipofectamine LTX reagent. Then, 48 h post transfection, cells were harvested, lysed, and supernatant was collected. Then, 100 µL of supernatant was loaded into the well of a black optiplate, to which 150 µL of luciferase assay buffer containing 25 mM glycylglycine (pH 7.8), 15 mM pottassium phosphate, 15 mM MgSO4, 4 mM EGTA, 2 mM ATP, 1 mM DTT, and 0.5 mM Luciferin was added, and luminescence was measured immediately using a multimode plate reader (PerkinElmer). Then, the luminescence units were normalized to total cellular protein.

### 2.9. RNA Extraction and Real-Time PCR Analysis of Target Genes

Total RNA from cells cultured under different treatment conditions were isolated using TRIzol method and quantified by Nanodrop spectrophotometer (Thermo scientific). First, 0.5–1 μg of total RNA was used for cDNA preparation using a cDNA synthesis kit (Applied biosystems) containing random hexamers, 10× RT buffer, and Multi Scribe reverse transcriptase in a 20 μL reaction system. Then, the cDNA was diluted in a ratio of 1:10 followed by Real-Time PCR in Roche light cycler 480 using SYBR Green chemistry. Appropriate internal control and non-template controls were included in all experiments. Melt curve analysis was carried out at the end of each run for checking the specificity of primers. The primers used were custom designed. The nucleotide sequences of the primers are listed below. The sequence of primers used is as follows.

ACTIN sense primer—5′-CCAACCGCGAGAAGATGA-3′
Anti-sense primer—5-CCAGAGGCGTACAGGGATAG-3′
APOL6 sense primer—5-AGTGAGGCTGGTGTTGGTTT-3′
Anti-sense primer—5′-CGTCTTGTAGCTCCACGTCTT-3′

### 2.10. Transfection of Cells

Hela cells at a confluency of 70% were transfected using polyethyleneimine reagent with 4 μg (6 well plates) of appropriate plasmid with GFP reporter vectors. After 12 h of incubation with the transfection mix, the medium was changed and replaced with fresh complete medium, and the cells were incubated further for 24 h. To determine the efficiency of transfection, cells were observed for green fluorescence under a fluorescent microscope (Leica).

### 2.11. Western Blotting Analysis

Cells grown under different treatment conditions were harvested and lysed in RIPA buffer followed by sonication. The supernatant collected was mixed with 6× SDS PAGE loading dye and heated at 90 °C for 10 min. Protein normalised samples were separated on 10% SDS PAGE and transferred on to PVDF membrane in a Trans blot apparatus. Then, the membrane was blocked with 5% BSA for 1 h at room temperature and incubated with primary antibody overnight at 4 °C. After overnight incubation, the membranes were washed with TBST and incubated with HRP-conjugated secondary antibody for 1 h. Then, the blots were developed using chemiluminescent reagent, and bands were visualised and quantified in a Versa Doc imaging system (BioRad Laboratories, Berkley, CA, USA).

### 2.12. Comet Assay

DNA fragmentation associated with apoptosis was determined by comet assay. Hela cells under different experimental conditions exposed to 25 µM cisplatin for 24 h, at a concentration of 10^5^ cells/mL, were mixed with 1% low melting agarose at a ratio of 1:3. Then, this suspension was evenly spread over a glass slide and allowed to set. After gelling, slides were placed in lysis solution (2% SDS, 0.5 M Na_2_EDTA, 0.5 mg/mL proteinase K at pH −8) and incubated at 37 °C overnight. After overnight incubation, slides were equilibrated in TAE solution and electrophoresed for 20 min at 12 V and 400 mA. Then, slides were stained with propidium iodide solution (20 mg/mL) for 20 min and observed under fluorescent microscope. The image analysis and quantification of the DNA damage in terms of olive tail moment was performed using open comet software available at www.cometbio.org (accessed on 15 November 2018).

### 2.13. Cytoplasmic and Mitochondrial Fractionation

Cytoplasmic and mitochondrial fractions were obtained according to the method described earlier [36]. Briefly, cells cultured under different experimental conditions were harvested and washed 3 times using ice-cold PBS and pelleted down by centrifuging at 200× *g* for 7 min. Then, the pellets were resuspended in 500 μL of STM buffer (250 mM sucrose, 50 mM Tris–HCl pH 7.4, 5 mM MgCl_2_, protease inhibitor cocktail) and incubated on ice for 30 min. After incubation, samples were vortexed at maximum speed for 15 s and centrifuged at 800× *g* for 15 min. The supernatant obtained was again centrifuged at 800× *g* for 10 min and to remove pellet followed by centrifugation at 11,000× *g* for 10 min to pellet down mitochondrial fraction. The supernatant from this step was precipitated using ice cold 100% acetone for 1 h followed by centrifugation at 12,000× *g* for 5 min. Thus, the pellet obtained was used as the cytoplasmic fraction, which was then resuspended in 200 μL STM buffer. The mitochondrial pellet obtained in the above step was resuspended in 200 μL of STM buffer and centrifuged at 11,000× *g* for 10 min. The supernatant was discarded, and the pellet was resuspended in 100 μL of SOL buffer (50 mM Tris–HCl pH 6.8, 1 mM EDTA, 0.5% Triton X-100 and protease inhibitor cocktail) and sonicated for 10 s at 70% amplitude with 15 s on and 30 s off cycles at 4 °C. Then, the samples were centrifuged at 12,000 rpm for 10 min at 4 °C, protein was estimated in the supernatant, and protein equivalent samples were prepared for SDS-PAGE analysis. The purity of the mitochondrial and cytoplasmic fractions were checked by analysing the presence of specific markers using Western blot analysis.

### 2.14. Statistical Analysis

Data were expressed as mean ± standard deviation. Duncan’s One-Way Analysis of Variance (ANOVA) was used to check the statistical significance of difference. All statistical analysis were performed using the SPSS 11.0 software. Differences were considered significant when *p* < 0.05.

## 3. Results

### 3.1. Expression Levels and Target Prediction Analysis of Some Candidate miRNAs in Exosomes Released from Cp-r HepG2 Cells

**Figure 1:**
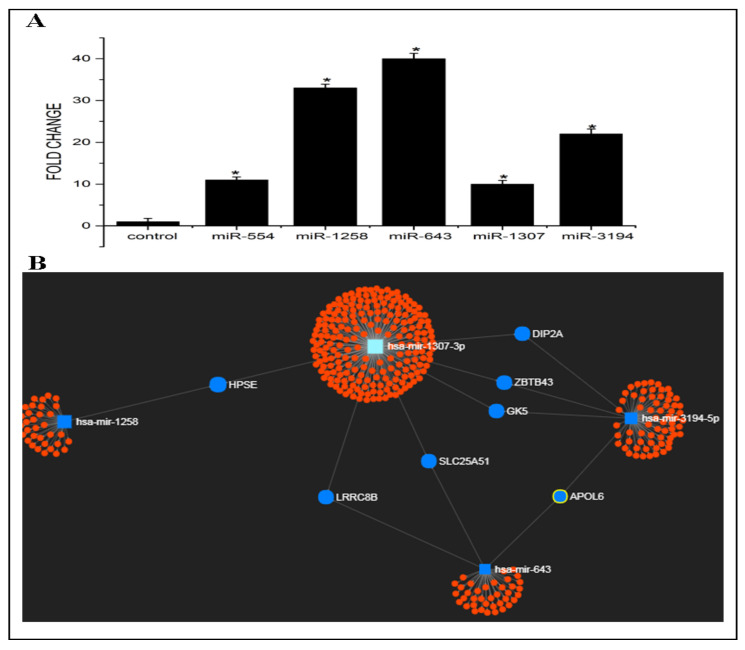
Expression levels and target prediction analysis of candidate miRNAs in exosomes secreted by Cp-r HepG2 cells (EXres). (**A**). Expression levels of candidate miRNAs in EXres. miRNAs were isolated from EXsen and EXres followed by cDNA synthesis and RT-PCR analysis of candidate miRNAs such as miR-554, miR-1258, miR-643, miR-1307, and miR-3194. The term fold change refers to the relative expression level of candidate miRNAs. Results presented are average of three experiments ± SEM each done at least in triplicate, *p* < 0.05. * Statistically significant when compared to control. (**B**). Target prediction analysis of candidate miRNAs. The hairball network represents candidate miRNAs targeting multiple genes. The centre part of the hairball structure represents the individual miRNA and edges represent their target genes. The genes that are commonly targeted by the candidate miRNAs are represented in blue colour.

The expression pattern of some candidate exosomal miRNAs such as miR-554, miR-1258, miR-643, miR-1307, and miR-3194, which are scarcely reported in tumour progression and metastasis so far, and not yet reported in drug resistance was analysed by RT-PCR. In order to check the level of these candidate miRNAs in exosomes released from Cp-r HepG2 cells, exosomal miRNAs were isolated from cisplatin-resistant and -sensitive HepG2 cells followed by cDNA synthesis and real-time PCR analysis. The results obtained showed that the level of all five candidate miRNAs was found to be significantly high in exosomes secreted by Cp-r HepG2 cells (EXres) when compared to that in exosomes secreted by Cp-s HepG2 cells (EXsen). Among these candidate miRNAs, miR-643 was found to be most significantly increased in EXres with a fold change of 40 when compared to EXsen (Figure 1A).

Target prediction analysis of candidate miRNAs such as miR-643, miR-554, miR-1258, miR-3194, and miR-1307 was performed using miRNet, which revealed that the network of these candidate miRNAs could target 415 genes. We next identified the genes that are commonly targeted by these miRNAs and checked the functions of these common target genes. Seven genes were found to be commonly targeted by this set of miRNAs, which include LRRC8B, SLC25A51, APOL6, ZBTB43, HPSE, GK5, and DIP2A. Among the candidate miRNAs, miR-643, which showed the highest fold difference, could target some important genes such as LRRC8B, SLC25A51, and APOL6, and among these genes, APOL6 was reported to be involved in apoptosis and may have a role in drug resistance (Figure 1B).

### 3.2. KEGG Pathway and GO Analysis of Candidate miRNAs

**Figure 2:**
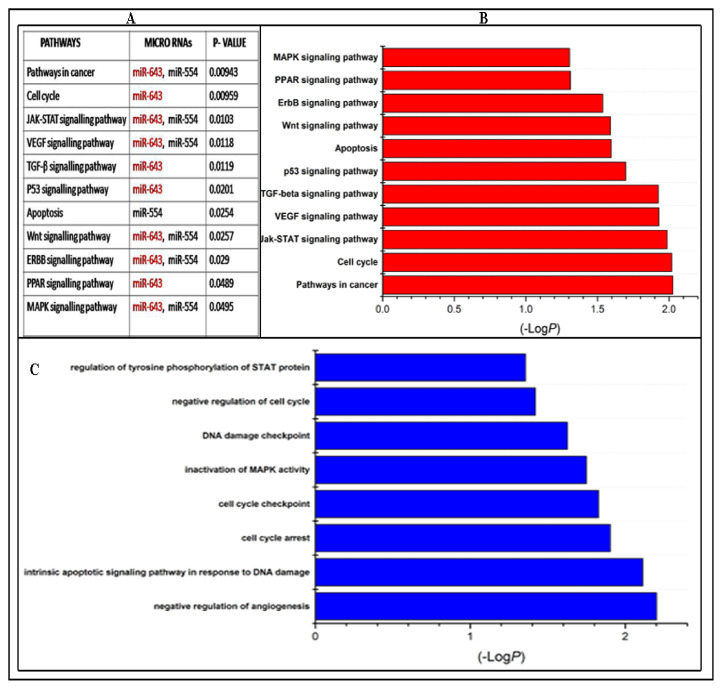
Pathway and GO analysis of candidate miRNAs. Pathway and GO analysis was performed using online web-based tool miRNet. (**A**). Pathways associated with the candidate miRNAs. (**B**). KEGG pathway analysis of candidate miRNA targets. Eleven pathway categories (*p* < 0.05) were significantly affected by five candidate miRNAs and five pathway categories were most significantly enriched. The y-axis represents the –log *p*-value and the x-axis represents different pathways. (**C**). GO analysis of candidate miRNA targets. Eight GO terms (*p* < 0.05) were significantly affected by candidate miRNAs and two GO terms were most significantly enriched. The *y*-axis represents −log *p*-value and the x-axis represents GO categories.

In order to characterise the predominant pathways associated with five candidate miRNAs such as miR-643, miR-554, miR-1258, miR-3194, and miR-1307, KEGG pathway enrichment analysis was performed. Pathway analysis revealed that a total of 100 pathways (*p* < 0.1) were associated with these candidate miRNAs. Among these 100 pathways, 11 pathway categories associated with cell proliferation or apoptosis were affected by these candidate miRNAs and were found to be enriched with a *p*-value of <0.05. However five pathway categories such as pathways in cancer, cell cycle, Jak-stat signalling pathway, VEGF signalling pathway, and TGF-β signalling pathways were most enriched and reported to be predominantly involved in drug resistance (Figure 2B).

GO analysis of five candidate miRNAs revealed that eight significant GO categories (*p* < 0.05) belonging to biological as well as molecular functions were regulated by candidate upregulated miRNA targets. However, only two GO categories such as intrinsic apoptotic signalling pathway in response to DNA damage and negative regulation of angiogenesis were significantly enriched, and all of these terms are directly or indirectly related with drug resistance (Figure 2C).

Altogether, these results suggests that miR-643 being the most significantly upregulated miRNA is predominantly involved in all the pathways obtained as hits in the algorithm and one of the target genes of miR-643, i.e., APOL6 is reported to be involved in mitochondrial apoptosis may have a role in drug resistance. Hence, we selected miR-643 for further validation.

### 3.3. Overexpression of miR-643 in Hela Cells

**Figure 3:**
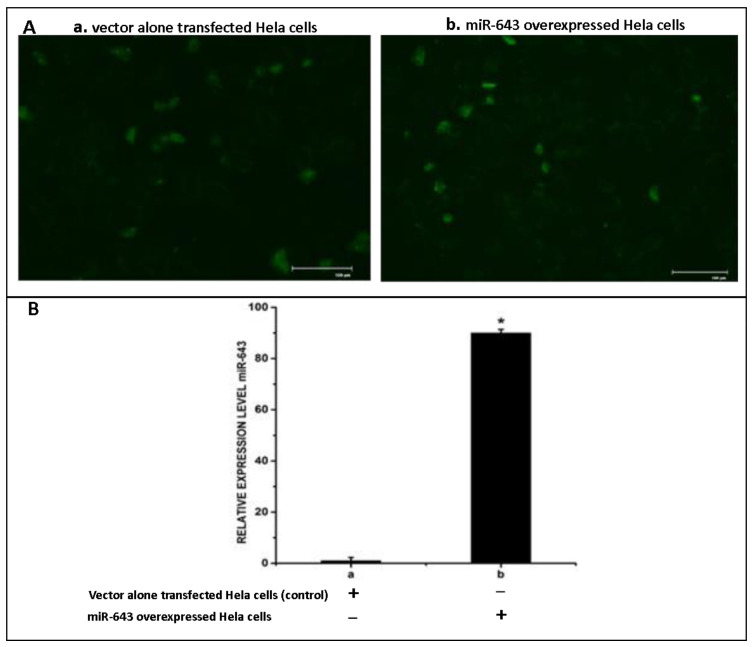
Overexpression of miR-643 in Hela cells. The construct expressing miR-643 was transfected into Hela cells, followed by RT-PCR analysis of miR-643 in transfected cells. (**A**) Microphotographs of cells transfected with **a**. pCMVmiR (vector control) **b**. miR-643 construct (**B**) Expression level of miR-643. In (B), a—pCMVmiR (vector control) transfected cells, b—miR-643 overexpressed cells. The mean values of two different experiments ± SEM each performed in triplicate are reported in the graph, *p* < 0.05. * indicates statistical significance when compared to a.

Subsequent to our observations that miR-643 was found to be at significantly higher levels in EXres, and only miR-643 was associated with all the pathways analysed, we next tried to validate the role of miR-643 in conferring drug resistance. Based on our previous reports that exosomes released from cisplatin-resistant HepG2 (EXres) cells can make the recipient cells more resistant to cisplatin, and that the level of miR-643 was found to be significantly higher in EXres, experiments were carried out to analyse if ectopic expression of miR-643 can modulate the chemo-sensitivity properties of recipient cells. For this, miR-643 was first cloned into miRNA cloning vector, pCMVmiR, and its expression was checked in Hela cells. The transfection efficiency was checked by visualising under a fluorescent microscope. The efficiency of transfection was found to be 75%. Furthermore, the expression level of miR-643 was determined by qRT-PCR, and the results suggested a 90-fold increase in the expression level of miR-643 in miR-643 transfected cells when compared to mock-transfected cells (Figure 3).

### 3.4. Exosomally Transferred miR-643 Can Modulate Cisplatin Sensitivity of Recipient Hela Cells

**Figure 4:**
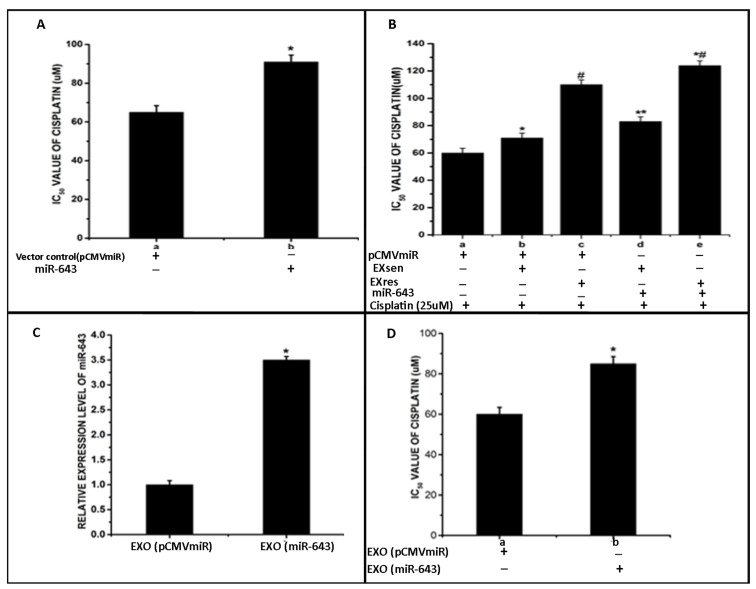
Exosomally conferred miR-643 can modulate cisplatin sensitivity of recipient Hela cells A. XTT viability assay carried out in the presence of miR-643-enriched exosomes and IC_50_ was determined. In (**A**), a—Vector alone transfected cells and b—miR-643 overexpressing cells. (**B**) Ectopic expression of miR-643 can augment the resistance conferred by EXsen/EXres. XTT assay was carried out in miR-643 overexpressing cells in presence or absence of EXsen/EXres. In B, a—mock-transfected cells + cisplatin (25 µM), b—mock-transfected cells + EXsen and cisplatin (25 µM), c—mock-transfected cells + EXres and cisplatin (25 µM), d—miR-643 overexpressing cells + EXsen and cisplatin (25 µM), e—miR-643 overexpressing cells + EXres and cisplatin. (**C**) qRT-PCR analysis of miR-643 levels in exosomes extractedfrom miR-643 overexpressing Hela cells. In (**C**), a—represents exosomes released from vector alone transfected cells, b—exosomes released from miR-643 overexpressing cells. (**D**) Exosomal delivery of miR-643 confers resistance properties to recipient Hela cells against cisplatin. XTTassay was carried out in Hela cells pretreated with 100 µg of miR-643 enriched exosomes or vector control exosomes In (**D**), a—Hela cells treated with exosomes released from mock-transfected cells (EXO_pCMVmiR_), b represents Hela cells treated with exosomes secreted from miR-643 overexpressing cells (EXO_miR-643_). The mean values of two different experiments ± SEM each performed in triplicate are reported in the graph, *p* < 0.05. * indicates statistical significance when compared to a. # indicates statistical significance when compared to a and b. ** indicates statistical significance when compared to b *# indicates statistical significance when compared to c.

Following our observation that miR-643 is found at the highest levels in EXres, in order to check the effect of ectopic expression of miR-643 on chemosensitivity of Hela cells, drug sensitivity assay was carried out in miR-643 overexpressing Hela cells. The results obtained showed cells expressing miR-643 to be more resistant towards cisplatin when compared to vector alone transfected cells, suggesting the role of miR-643 in cisplatin resistance (Figure 4A). Drug sensitivity assay was carried out to check the effect of exosomes released from Cp-s (EXsen) and Cp-r (EXres) HepG2 cells on the cisplatin sensitivity properties of Hela cells, which showed that EXres containing higher levels of miR-643 could make the cells resistant to cisplatin, as provided in Figure 1B(ii) of Raji et al., 2017, and the ectopic expression of miR-643 as discussed above desensitised the cells to cisplatin, we next analysed if cisplatin resistance conferred in response to EXres could be further increased by ectopic expression of miR-643. For this, miR-643 overexpressing Hela cells were treated with EXsen or EXres followed by XTT viability assay. XTT assay results showed that miR-643 overexpressing cisplatin-sensitive cells treated with EXres became resistant to cisplatin, and an enhancing effect of miR-643 expression and EXres was observed, suggesting that ectopic expression of miR-643 could augment cisplatin resistance conferred in response to EXres. Furthermore, increased resistance to cisplatin was observed in miR-643 overexpressing cells treated with EXsen (Figure 4B).

In continuation to this observation, we next checked whether exosomes containing increased levels of miR-643 can bring the same effect. For this, exosomes were isolated from miR-643 overexpressing Hela cells, and the presence of exosomes was confirmed by checking exosomal CD63 markers using ELISA followed by RT-PCR analysis to check the levels of miR-643 in these exosomes. The results obtained showed about 2–3 fold higher levels of miR-643 in exosomes released from miR-643 overexpressing cells (EXO_miR-643_) in comparison with exosomes released from vector alone transfected cells (EXO_pCMVmiR_) (Figure 4C). Results of XTT viability assay carried out in Hela cells, in the presence of these exosomes, showed that miR-643 enriched exosome treated cells became resistant to cisplatin, suggesting that the exosomal delivery of miR-643 can desensitise the cells towards cisplatin (Figure 4D).

### 3.5. EXres Mediated Alteration in Cisplatin Resistance Involving APOL6

**Figure 5:**
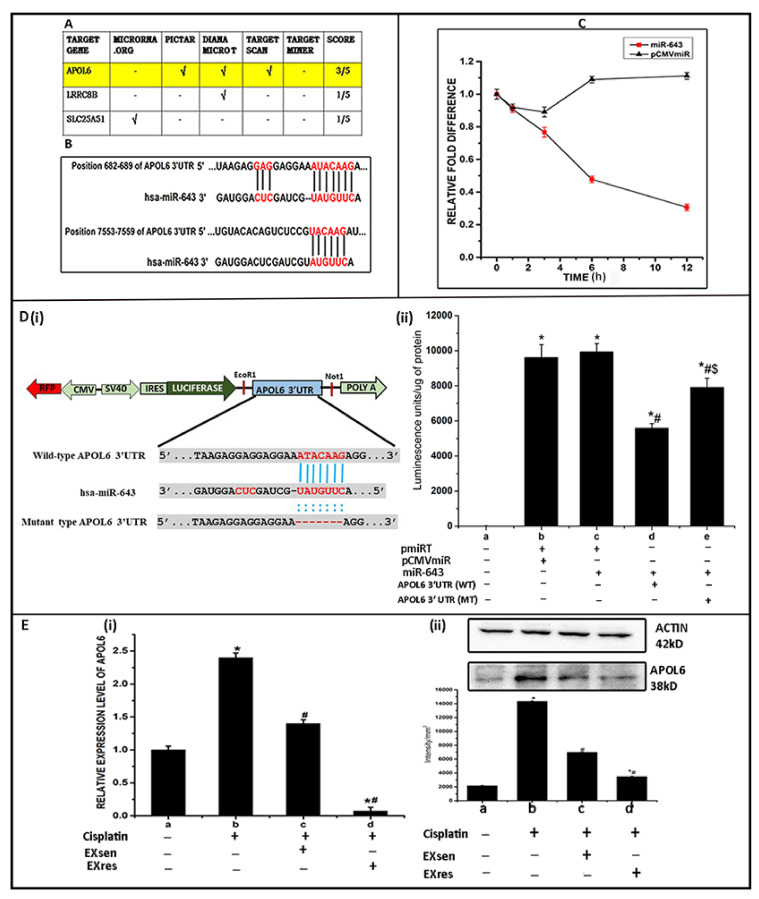
EXres-mediated alteration in cisplatin resistance involves APOL6. (**A**) Target prediction analysis of miR-643. Target prediction analysis of miR-643 was carried out using five different web-based tools such as microrna.org, Pictar, Diana micro T, TargetScan, and Target miner followed by scoring. (**B**) 3′UTR analysis of APOL6 was carried out using a web-based tool TargetScan showing the sequence alignments between miR-643 and 3′UTR of APOL6. (**C**) APOL6 mRNA stability assay. miR-643 overexpressing cells were treated with actinomycin at a concentration of 6 µg for different time intervals such as 0, 1, 3, 6, and 12 h followed by RNA isolation from these cells and RT-PCR analysis of APOL6 mRNA. The term relative fold difference refers to relative expression level of APOL6 mRNA in the presence or absence of miR-643. (**D**) Luciferase reporter assay. The wild and mutant APOL6 3′UTR were cloned into pMIRTARGET reporter vector downstream of luciferase followed by luciferase activity assay. (**i**) Schematic representation of the modified luciferase vector containing 3′UTR of the APOL6 gene and sequence comparison among APOL6 wild-type 3′UTR, APOL6 mutant-type 3′UTR, and miR-643. (**ii**) APOL6 3′UTR luciferase assay results in Lum/µg of protein (**E**). EXres can modulate APOL6 mRNA and protein levels. APOL6 mRNA and protein levels were checked in Hela cells treated with EXsen/EXres and cisplatin. (**i**) qRT-PCR analysis of APOL6 mRNA. (**ii**) Levels of APOL6 protein analysed by Western blot. In (**i,ii**) a—untreated cells b—cells + cisplatin, c—cells + EXsen and cisplatin and d—cells + EXres and cisplatin. The mean values of two different experiments ± SEM each performed in triplicate are reported in the graph, *p* < 0.05. * indicates statistical significance when compared to a. # indicates statistical significance when compared to b. *# indicates statistical significance when compared to b and c. *#$ indicates statistical significance when compared to b, c and d.

Subsequent to our findings that miR-643 can augment cisplatin resistance in Hela cells, we further checked the underlying regulatory mechanism involved in rendering resistance. For this, targets of miR-643 predicted by miRNet were cross checked with five different web-based tools such as microrna.org, Diana micro T, TargetScan, Pictar, and Target miner followed by scoring of the identified targets. The target gene identified by at least three of the five independent tools was selected for further mechanistic study. Among the candidate targets, only APOL6 has attained our criteria with a score of 3/5 and was selected for further analysis (Figure 5A). Then, 3′UTR analysis of APOL6 was performed using the online algorithm available at TargetScan (http://www.targetscan.org (accessed on 10 December 2018)). The 3′UTR analysis results revealed that 3′UTR of APOL6 contains two putative regions at nucleotides 682–689 and 7553–7559 that match the seed sequence of miR-643, suggesting that 3′UTR of APOL6 possess potential binding sites for miR-643 (Figure 5B). In order to check whether APOL6 is functionally targeted by miR-643, APOL6 mRNA stability assay was carried out, and the results obtained showed that APOL6 mRNA stability was significantly reduced under miR-643 overexpression condition, suggesting that the regulatory role of miR-643 involves alterations of post-transcriptional stability of APOL6 mRNA (Figure 5C). The results of luciferase reporter assay further validated the interaction between miR-643 and APOL6. As shown in Figure 5D(ii), miR-643 could markedly decrease the luciferase reporter activity of the construct expressing wild-type APOL6 3′UTR, while for the mutant type, this repression was relieved, indicating that miR-643 has binding sites on the 3′UTR region of APOL6 mRNA.

APOL6, being identified as the potential target of miR-643, we next analysed if cisplatin resistance conferred byEXsen or EXres is mediated by APOL6. For this, we checked APOL6 mRNA and protein levels in Hela cells treated with EXsen/EXres and cisplatin. Results obtained showed that the levels of APOL6 mRNA and protein were significantly decreased in EXres treated cells when compared to EXsen and untreated cells, suggesting that EXres-mediated cisplatin resistance may involve APOL6 (Figure 5E).

### 3.6. Ectopic Expression of miR-643 Can Reduce APOL6 Levels and Augments the Effect of EXres on APOL6 Levels

**Figure 6:**
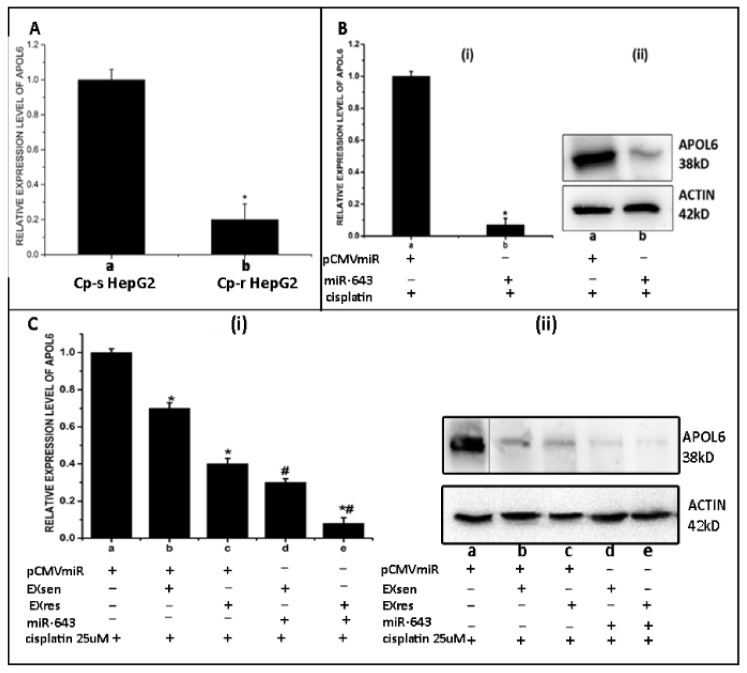
Ectopic expression of miR-643 can reduce APOL6 levels and augment the effect of EXres on APOL6 levels. (**A**) Expression level of APOL6 in Cp-r HepG2 cells. RNA was isolated from Cp-s and Cp-r HepG2 cells followed by cDNA synthesis and RT-PCR analysis of APOL6. In A, a represents Cp-s HepG2 cells and b represents Cp-r HepG2 cells. (**B**) APOL6 mRNA and protein levels under miR-643 overexpression condition. Hela cells were transfected with pCMVmiR (control) or miR-643 construct. 48 h post transfection, protein and RNA was isolated to estimate APOL6 expression levels by real-time PCR (**i**) and protein levels were analysed by Western blot analysis (**ii**). In (**B**), a—vector alone transfected cells + cisplatin (25 µM) and b—miR-643 overexpressed cells + cisplatin (25 µM). Results presented are the average of two experiments ± SEM each done at least in triplicate, *p* < 0.05. * Statistically significant when compared to a. (**C**) Ectopic expression of miR-643 augments the effect of EXres on APOL6 levels. miR-643 overexpressing Hela cells were treated with EXsen/EXres followed by analysis of APOL6 mRNA and protein levels. (**i**) APOL6 mRNA levels analysed by RT-PCR. (**ii**) APOL6 protein levels analysed by Western blot. In (**i**,**ii**) a—vector only transfected cells + cisplatin, b—vector only transfected cells + cisplatin and EXsen, c—vector only transfected cells + cisplatin and EXres, d—miR-643 overexpressing cells + cisplatin and EXsen and e—miR-643 overexpressing cells + cisplatin and EXres. The mean values of two different experiments ± SEM each performed in triplicate are reported in the graph, *p* < 0.05. * indicates statistical significance when compared to a and b. # indicates statistical significance when compared to b. *# indicates statistical significance when compared to c. Two irrelevant lanes were spliced out from the blot C (**ii**) of APOL6 and full-length blots are given in Figure S3.

Since it was observed that EXres mediated cisplatin resistance may involve APOL6, we next analysed the expression level of APOL6 in cisplatin-resistant HepG2 cells. For this, RNA was isolated from cisplatin-sensitive and cisplatin-resistant HepG2 cells followed by RT-PCR analysis of APOL6. RT-PCR results showed that the levels of APOL6 were significantly low in Cp-r HepG2 cells when compared to Cp-s cells (Figure 6A). Furthermore, we checked the effect of ectopic expression of miR-643 on APOL6 levels. For this, APOL6 mRNA and protein levels were checked in miR-643 overexpressing cells by RT-PCR and Western blot, and the results obtained showed that APOL6 mRNA and protein levels were significantly reduced in miR-643 overexpressing cells (Figure 6B).

Since it was observed that ectopic expression of miR-643 reduces APOL6 levels, we next checked the effect of ectopic expression of miR-643 on APOL6 levels in the presence of EXsen/EXres. For this, miR-643 overexpressing cells were treated with EXsen/EXres followed by analysis of the levels of APOL6 mRNA and protein. The results obtained showed that the levels of APOL6 were significantly reduced in miR-643 overexpressing cells treated with EXres/EXsen when compared to untreated control or EXsen/EXres alone treated cells, suggesting that miR-643 can augment the effect of EXres on APOL6 levels (Figure 6C).

### 3.7. miR-643-Mediated, EXres-Dependent Alteration in Cisplatin Resistance Involve Reduced Apoptosis Resulting in Promotion of Cell Survival

**Figure 7:**
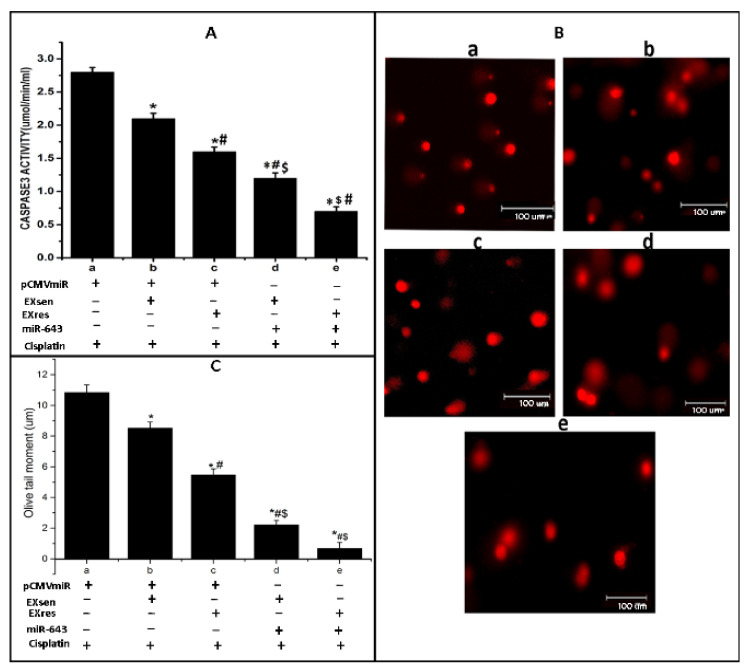
miR-643-mediated, EXres-dependent alteration in cisplatin resistance involves reduced apoptosis, resulting in promotion of cell survival. miR-643 overexpressing Hela cells were treated with EXsen/EXres in the presence of 25 µM cisplatin for 24 h followed by caspase3 activity assay and comet assay. (**A**). Caspase 3 activity assay. (**B**). Representative microphotographs of comets obtained from comet assay in miR-643 overexpressing Hela cells treated with EXsen/EXres. (**C**). Olive tail moment analysed by comet assay in miR-643 overexpressing Hela cells treated with EXsen/EXres. In (**A**–**C**), a—vector control + cisplatin, b—vector control + cisplatin+ EXsen, c—vector control + cisplatin + EXres, d—miR-643 overexpressing cells + cisplatin + EXsen and e—miR-643 overexpressing cells + cisplatin + EXres. The mean values of two different experiments ± SEM each performed in triplicate are reported in the graph, *p* < 0.05. * indicates statistical significance when compared to a. # indicates statistical significance when compared to b. $ indicates statistical significance when compared to c.

Subsequent to our finding that miR-643 can augment EXres-mediated cisplatin resistance, we next checked whether miR-643-mediated, EXres-dependent cisplatin resistance occurs by promoting cell survival or by altering cellular apoptosis. For this, apoptotic assays such as comet and caspase 3 activity assays were carried out in miR-643 overexpressing Hela cells treated with EXsen or EXres in the presence of cisplatin. The results obtained showed that caspase 3 activity was significantly lowered in miR-643 overexpressing cells treated with EXsen or EXres, in comparison with control (Figure 7A). Caspase 3 activity assay results were supported by comet assay results, which showed decreased apoptotic levels indicated by a reduced rate of DNA damage or fragmentation as evident by reduced olive tail moment in miR-643 overexpressing Hela cells treated with EXsen or EXres. These results clearly suggest that ectopic expression of miR-643 decreased the percentage of cells undergoing apoptosis, thus promoting cell survival (Figure 7B,C).

### 3.8. Exosomes Enriched with miR-643 Reduces APOL6 Levels, Decreases Caspase Activity, and Promotes Cell Survival in Recipient Cells

**Figure 8:**
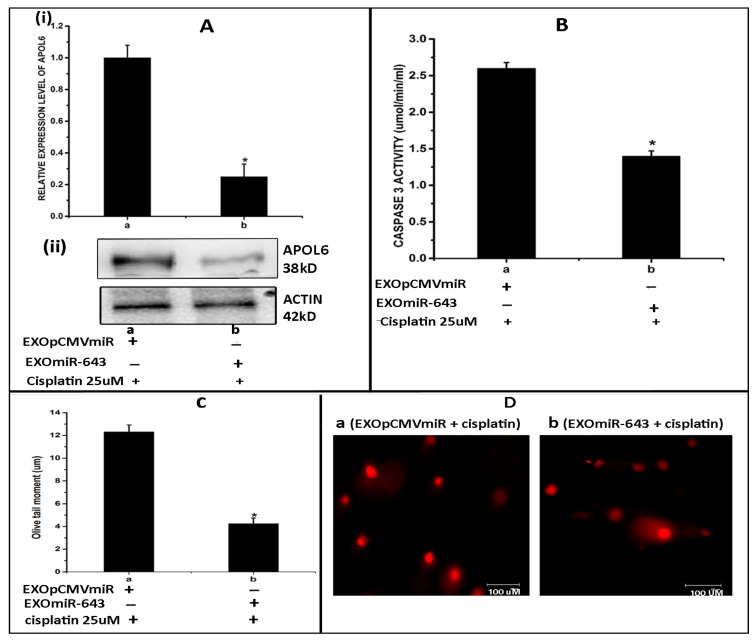
Exosomes enriched with miR-643 reduces APOL6 levels, decreases caspase activity, and promotes cell survival in recipient cells. Hela cells were treated with miR-643 enriched exosomes 24 h post treatment, RNA and protein was isolated to determine APOL6 mRNA and protein levels by RT-PCR and Western blot analysis, respectively. (**A**) Levels of (**i**) APOL6 mRNA analysed by RT-PCR (**ii**) APOL6 protein analysed by Western blot. (**B**) Caspase 3 activity in Hela cells treated with EXO_miR-643_. (**C**) Representative microphotographs of comets obtained from comet assay in Hela cells treated with EXO_miR-643_. (**D**) Olive tail moment analysed by comet assay in Hela cells treated with EXO_miR-643_ using open comet software. In (**A**–**D**), a—Hela cells treated with EXO_(pCMVmiR)_ and cisplatin and b—Hela cells treated with EXO_(miR-643)_ + cisplatin. The mean values of two different experiments ± SEM each performed in triplicate are reported in the graph, *p* < 0.05. * indicates statistical significance when compared to a.

Following our observation that miR-643 can modulate the expression pattern of APOL6 mRNA and protein levels, we further tried to check the effect of miR-643 enriched exosomes on APOL6 levels. For this, exosomes were isolated from miR-643 overexpressing and vector alone transfected Hela cells and increased levels of miR-643 were confirmed in exosomes secreted from miR-643 overexpressing cells by RT-PCR. Furthermore, Hela cells were treated with these exosomes followed by analysis of APOL6 mRNA and protein levels. The results obtained revealed that APOL6 mRNA and protein levels were significantly decreased in cells treated with exosomes containing higher levels of miR-643, suggesting that exosomal delivery of miR-643 could reduce APOL6 levels (Figure 8A).

Since it was observed that miR-643-mediated, EXres-dependent cisplatin resistance involves the modulation of apoptosis and promotion of cell survival, we next analysed the effect of miR-643-enriched exosomes on cell survival. For this, apoptotic assays such as caspase 3 activity and comet assay was carried out in Hela cells treated with miR-643 enriched exosomes. Results obtained showed that caspase 3 activity was significantly lowered in cells treated with an miR-643-enriched exosome when compared to control cells (Figure 8B). Results of comet assay also showed a decreased extent of DNA damage or fragmentation as evident by reduced olive tail momentin cells treated with EXO_miR-643_ (Figure 8C,D). Thus, these results suggest that the exosomal delivery of miR-643 can reduce caspase 3 activity and promote cell survival by reducing APOL6 levels.

### 3.9. miR-643-Mediated Promotion of Cell Survival and Cisplatin Resistance Is APOL6 Dependent

**Figure 9:**
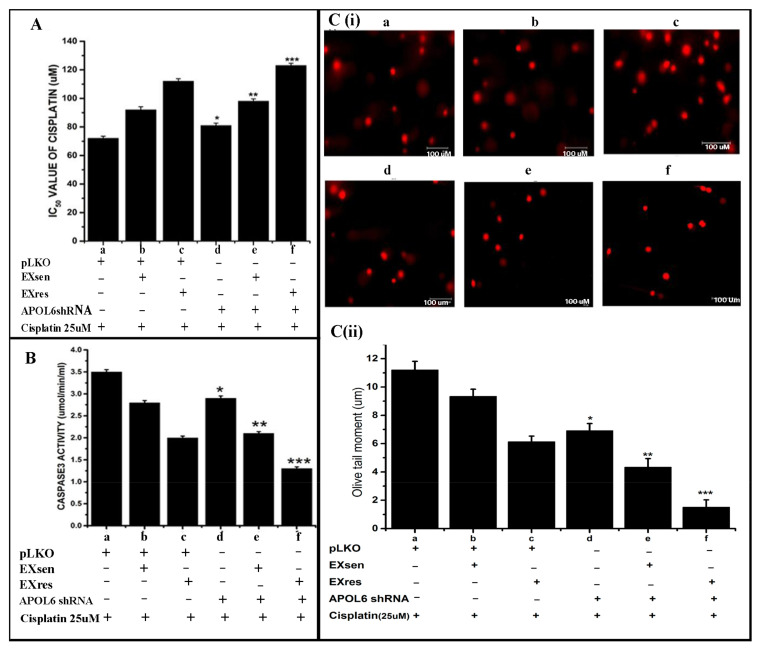
Effect of APOL6 knockdown on EXres-dependent alteration in cisplatin resistance. APOL6 knocked down cells were exposed to EXsen or EXres and cisplatin (25 µM) followed by XTT assay, comet, and caspase3 activity assay. (**A**) XTT assay. (**B**) Caspase3 activity assay (**C**) (**i**) Representative microphotographs of comets and (**C**) (**ii**) Olive tail moment analysed by comet assay using open comet software. In (**A**–**C**), a represents pLKO vector control + cisplatin, b represents pLKO vector control + EXsen + cisplatin, c represents pLKOvector control + EXres + cisplatin, d represents APOL6 shRNA + cisplatin, e represents APOL6 shRNA + EXsen + cisplatin, and f represents APOL6 shRNA + EXres + cisplatin. The mean values of two different experiments ± SEM each performed in triplicate are reported in the graph, *p* < 0.05. * indicates statistical significance when compared to a. ** indicates statistical significance when compared to b. *** indicates statistical significance when compared to c.

**Figure 10:**
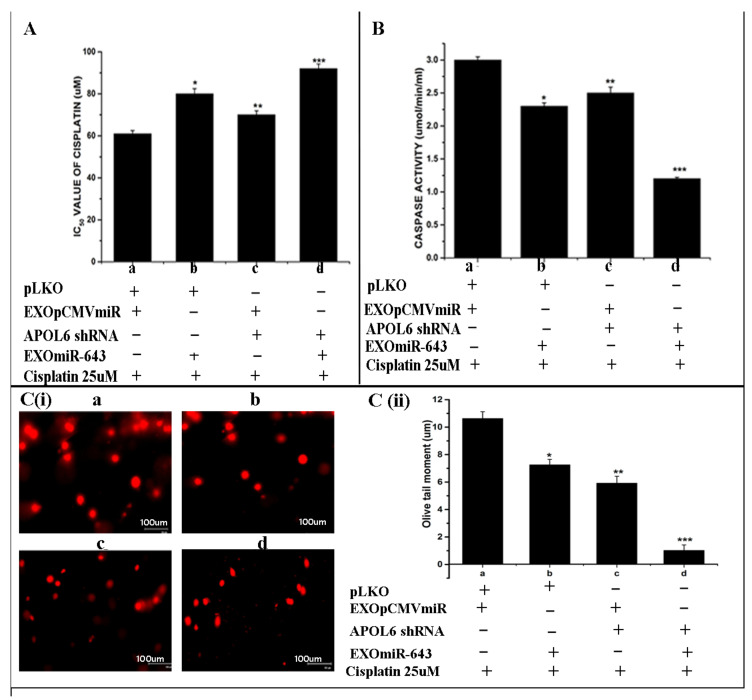
Effect of APOL6 knockdown on miR-643-mediated alteration in cisplatin resistance. APOL6 knocked down cells were treated with 100 µg of miR-643 exosomes in the presence of cisplatin (25 µM) followed by XTT assay, comet and caspase 3 activity assay. (**A**) Cytotoxicity assay (**B**) Caspase 3 activity assay (**C**) (**i**) Representative microphotographs of comets (**C**) (**ii**) Levels of DNA damage analysed by comet assay using opencomet software. In (**A**–**C**), a—represents pLKO vector control + EXO_(pCMVmiR)_ + cisplatin, b—represents pLKO vector control + EXO_miR-643_+ cisplatin, c—represents APOL6 shRNA + EXO_pCMVmiR_ + cisplatin, d—represents APOL6 shRNA + EXO_miR-643_ + cisplatin. The mean values of two different experiments ± SEM each performed in triplicate are reported in the graph, *p* < 0.05. * indicates statistical significance when compared to a. ** indicates statistical significance when compared to b. *** indicates statistical significance when compared to c.

On account of our findings that (a) miR-643 can modulate the sensitivity properties of Hela cells towards cisplatin, (b) EXres-mediated acquisition of cisplatin resistance in Hela cells is due to an increase in miR-643, (c) APOL6 is a major target of miR-643, and (d) miR-643 can augment the effect of EXres on APOL6 levels and promote cell survival, we next tried to confirm if the modulation in cisplatin resistance mediated by EXres is dependent on APOL6 levels. For this, APOL6 knockdown assays were done. Results of drug sensitivity assay and apoptotic assays such as caspase3 activity and comet assay carried out under APOL6 knocked down conditions with or without EXsen/EXres or miR-643 exosomes showed that knockdown of APOL6 in cells treated with EXres or miR-643 exosomes conferred a greater degree of resistance against cisplatin. Along with these results, the decreased caspase3 activity and the decreased rate of DNA damage observed in EXres or miR-643 exosome-treated cells suggest that miR-643-mediated alteration in cisplatin resistance is dependent on APOL6 (Figure 9 and Figure 10).

### 3.10. miR-643-Mediated Modulation of Cisplatin Resistance in Hela Cells Is Dependent on APOL6-Induced Release of Cytochrome C

**Figure 11:**
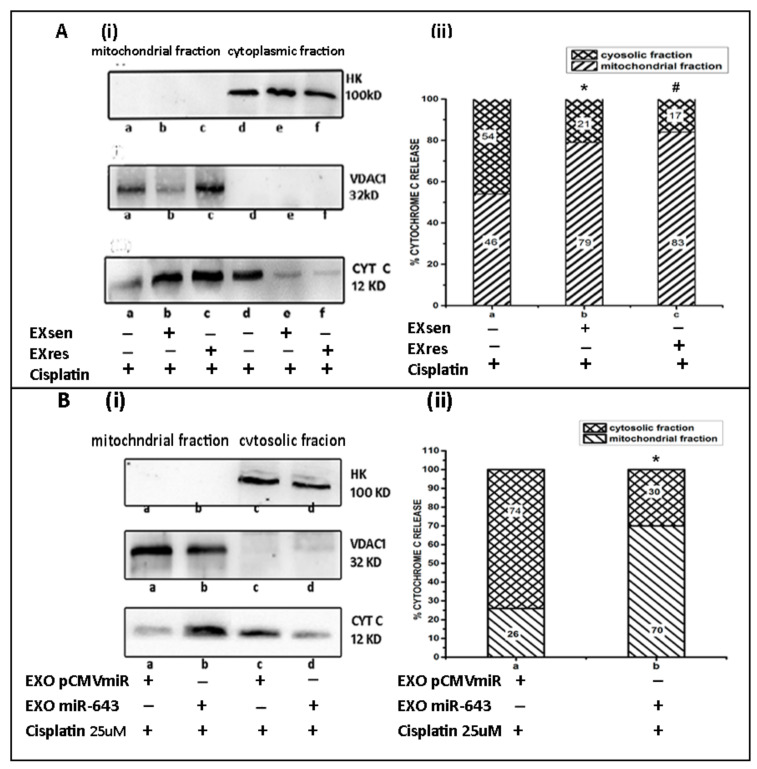
miR-643-mediated modulation of cisplatin resistance in Hela cells is dependent on APOL6-induced release of cytochrome C. (**A**) (**i**) Levels of HK, VDAC1, and CYT C proteins analysed by Western blot from mitochondrial and cytplasmic fractions of Hela cells treated with EXsen/EXresand cisplatin. In (**A**) (**i**), a—mitochondrial fractions of cells exposed to cisplatin, b—mitochondrial fractions of cells exposed to EXsen + cisplatin and c—mitochondrial fractions of cells exposed to EXres +cisplatin, d—cytosolic fraction of cells exposed to cisplatin, e—cytosolic fraction of cells exposed to EXsen + cisplatin and f—cytosolic fraction of cells exposed to EXres + cisplatin. (**ii**). The relative proportion of cytosolic to mitochondrial cytochrome c from EXsen/EXres and cisplatin-treated Hela cells was calculated and presented in graphical form. In (**A**) (**ii**) a—cells exposed to cisplatin, b—cells exposed to EXsen + cisplatin and c—cells exposed to EXres + cisplatin. (**B**) (**i**) Levels of HK, VDAC1, and CYT C proteins analysed by Western blot from mitochondrial and cytplasmic fractions of Hela cells treated with EXO_miR-643_. In (**B**) (**i**) a—mitochondrial fraction of Hela cells exposed to EXO_pCMVmiR_ and cisplatin, b—mitochondrial fraction of Hela cells exposed to EXO_miR-643_ and cisplatin, c—cytosolic fraction of Hela cells exposed to EXO_pCMVmiR_ and cisplatin, d—cytosolic fraction of Hela cells exposed to EXO_miR-643_ and cisplatin. (**ii**) The relative proportion of cytosolic to mitochondrial cytochrome c from Hela cells treated with EXO_miR-643_ was calculated and presented in graphical form. In (**C**) (**ii**) a—Hela cells exposed to EXO_pCMVmiR_ and cisplatin, b—Hela cells exposed to EXO_miR-643_ and cisplatin. The mean values of two different experiments ± SEM each performed in triplicate are reported in the graph, *p* < 0.05. * indicates statistical significance when compared to a. # indicates statistical significance when compared to b.

APOL6 has been reported to play an important role in mitochondria-mediated apoptosis by inducing the release of cytochrome c from mitochondria. In order to check the role of miR-643 in altering cisplatin resistance by regulating APOL6-dependent cell death, levels of cytosolic and mitochondrial cytochrome c protein were checked in EXsen/EXres and cisplatin-treated cells. For this, cytoplasmic and mitochondrial fractions were generated from EXsen/EXres and cisplatin-treated Hela cells. The purity of cytoplasmic and mitochondrial fractions was analysed by checking the presence of cytoplasmic marker hexokinase (HK) and mitochondrial marker, VDAC1 followed by analysis of Cyt c levels in both the fractions. The results obtained showed that the cytoplasmic release of cytochrome c protein was significantly reduced in cells treated with EXres when compared to EXsen-treated cells (Figure 11A), thus indicating a possible interaction of APOL6 with cytochrome c leading to cisplatin resistance being conferred in response to EXres. Then, experiments were carried out to check cytochrome c levels in mitochondrial and cytosolic fractions of cells treated with exosomes containing higher levels of miR-643. The cytoplasmic release of cytochrome c was also found to be significantly lower in cells treated with EXO_miR-643_ when compared to control cells (Figure 11B), indicating the role of miR-643 in making the cells more resistant to cisplatin by preventing APOL6-mediated release of cytochrome c and hence apoptosis.

## 4. Discussion

Acquired resistance to cisplatin is a major hurdle for the success of cisplatin-based combination therapies for cancer [37]. Therefore, intense investigation into the underlying mechanisms of acquisition of cisplatin resistance at cellular and molecular levels is essential, which can contribute to effective therapies that can combat chemoresistance [38]. Recent studies suggest that the selective modulation of the levels of exosomal miRNAs can boost the response to chemotherapy by alleviating the acquisition of drug resistance [39]. Many studies have evaluated the importance of exosomes in cancer progression. Previous studies including ours have revealed that drug-resistant tumour cells are an ample source of exosomes that can serve as paracrine modulators via the horizontal transfer of miRNAs [40]. Multiple studies have reported the role of tumour derived exosomal miRNAs in conferring drug resistance to recipient cells [41]. However, detailed studies on the exosomal miRNA expression pattern responsible for chemosresistance is still lacking. In view of these facts, we tried to check the expression pattern of some candidate exosomal miRNAs, which are less reported in tumour progression and metastasis so far, and not reported yet in drug resistance from cisplatin-resistant HepG2 cells, and functionally validate the role of most significantly dysregulated miRNA in drug resistance. The candidate miRNAs analysed include miR-554, miR-1258, miR-643, miR-1307, and miR-3194. Expression analysis of candidate miRNAs revealed miR-643 to be the most abundant miRNA in EXres. Furthermore, pathway and GO analysis of these candidate miRNAs showed that among the five candidate miRNAs subjected to pathway analysis using miRNet and miRsystem, miR-643 was found to be associated with all the 11 pathway categories analysed. Since miR-643 is the most significantly upregulated miRNA and is involved in all the 11 pathways analysed, we next predicted the possible targets of miR-643. One of the target genes of miR-643 as per the target prediction algorithm was APOL6 which has been reported to be involved in mitochondrial apoptosis. Taking into consideration the facts that miRNAs packaged in the form of exosomes can modulate the drug resistance properties of recipient cells against that drug, and previous data from our lab that exosomes from cisplatin-resistant (Cp-r) HepG2 cells can impart cisplatin resistance properties to recipient Hela cells [24], the present study was designed to identify the role of exosomal miR-643 secreted by Cp-r Hepatocarcinoma (HepG2) cells in altering the drug-resistance properties of recipient cervical cancer (Hela) cells towards cisplatin. A previous study involving the profiling of high-density lipoprotein (HDL) associated microRNA showed that miR-643 is involved in HDL-mediated transportation [42]. Several studies have reported the tumour-suppressive role of miR-643 [33,43]. So far there are no data available for the oncogenic role of miR-643, particularly in drug resistance. In order to check the role of horizontal transfer of miR-643 in cisplatin resistance, miR-643 was cloned and overexpressed in Hela cells. Our previous reports have shown that EXres can make the recipient Hela cells resistant towards cisplatin, and based on the fact that EXres contain a higher level of miR-643, we designed experiments to check if higher levels of miR-643 in EXres could contribute to cisplatin resistance. For this, the effect of ectopic expression of miR-643 in altering cisplatin resistance of Hela cells was analysed. The results obtained showed that miR-643-overexpressing cells became more resistant to cisplatin, demonstrating an important role of miR-643 in cisplatin resistance. Furthermore, we checked if ectopic expression of miR-643 could augment EXsen/EXres-mediated cisplatin resistance, and the results obtained showed that miR-643 overexpressing Hela cells treated with EXres became resistant to cisplatin, suggesting that ectopic expression of miR-643 could augment cisplatin resistance conferred in response to EXres. Next, we analysed if exosomal delivery of miR-643 could confer resistance properties to cells towards cisplatin. For this, cisplatin-sensitive Hela cells were treated with miR-643 enriched exosomes, which made them more resistant to cisplatin assisting our earlier observation of miR-643 as an important modulator of cisplatin resistance mediated by EXres. Our results were consistent with previous reports suggesting that exosomes from cisplatin-exposed lung cancer cells containing higher levels of miR-21 could confer resistance to recipient cells [44]. Following our findings that horizontal transfer of miR-643 can render cisplatin resistance to recipient cells, we further aimed to check the underlying mechanism that modulates cisplatin resistance. For this, targets of miR-643 predicted by a web-based tool miRNet as described earlier were cross-checked using multiple miRNA analysis tools and scored. Out of the several targets, APOL6 was chosen for further functional analysis, as scoring of the multiple targets of miR-643 indicated APOL6 as the highest scored target gene. Further, 3′UTR analysis of APOL6 mRNA was carried out using an online algorithm available at target scan, which revealed that 3′UTR of APOL6 mRNA possess two potential binding sites for miR-643. In order to check if the regulation of APOL6 by miR-643 is mediated by binding of miR-643 to the 3′UTR of APOL6 mRNA, wild and mutant APOL6 3′UTR were cloned into pMIRTARGET reporter vector downstream of luciferase gene. Results of luciferase assay revealed that miR-643 could markedly decrease the luciferase activity of the wild type, while for the mutant type, this repression was relieved, indicating that miR-643 has binding sites on the 3′UTR region of APOL6 mRNA, as observed in in silico analysis. Furthermore, mRNA stability assay suggested that miR-643 can alter the post-transcriptional stability of APOL6 mRNA. APOL6 is a lipid-binding protein possessing BH3 domain, and it has been reported to mediate apoptosis by interacting with Bcl-2 family members [45,46]. Furthermore, to identify if APOL6 has any role in miR-643-mediated cisplatin resistance, APOL6 mRNA and protein levels were analysed in EXsen or EXres and cisplatin-treated Hela cells. APOL6 levels were found to be markedly decreased in EXres-treated cells when compared to that treated with EXsen and untreated control cells. We also checked if the levels of APOL6 can be altered by ectopic expression of miR643, and the results obtained showed significantly lower levels of APOL6 mRNA and protein in cells expressing miR-643 treated with EXsen/EXres, suggesting that miR-643 can augment the effect of EXres on APOL6 levels. Subsequent to our finding that miR-643 can augment EXres-mediated cisplatin resistance, we next checked whether miR-643-mediated cisplatin resistance occurs by promoting cell survival. For this, apoptotic assays such as comet and caspase 3 activity assays were carried out under the same experimental conditions, and the results obtained showed lowered caspase 3 activity and decreased levels of DNA damage in miR-643 overexpressing cells treated with EXsen/EXres, suggesting that ectopic expression of miR-643 imparts an additive effect to EXres mediated cisplatin resistance, thereby promoting cell survival. As miR-643 can modulate APOL6 levels, we further analysed the effect of miR-643 enriched exosomes on APOL6 levels. APOL6 mRNA and protein levels were markedly reduced in miR-643 enriched exosome treated cells. Furthermore, the treatment of cells with EXO_miR-643_ lead to lowered caspase 3 activity and a decreased rate of DNA damage, suggesting that the exosomal delivery of miR-643 promote cell survival by reversing APOL6-mediated apoptosis, similar to that of conditions of APOL6 knockdown.

APOL6 has been reported as a pro-apoptotic protein that triggers the secretion of cytochrome c into cytosol from inter-mitochondrial space, thus promoting apoptosis [47]. To further prove the role of miR-643 in APOL6-mediated apoptosis, experiments were designed to check the effect of EXsen/EXres or EXO_miR-643_ on the levels of cytoplasmic and mitochondrial cytochrome c. As expected, cytoplasmic release of cytochrome c was markedly reduced in EXres treated as well as EXO_miR-643_-treated cells in comparison with control cells, suggesting the role of miR-643/EXres in reversing APOL6-mediated apoptosis by preventing APOL6-induced release of cytochrome c, thus promoting cell survival.

## 5. Conclusions

**Figure 12:**
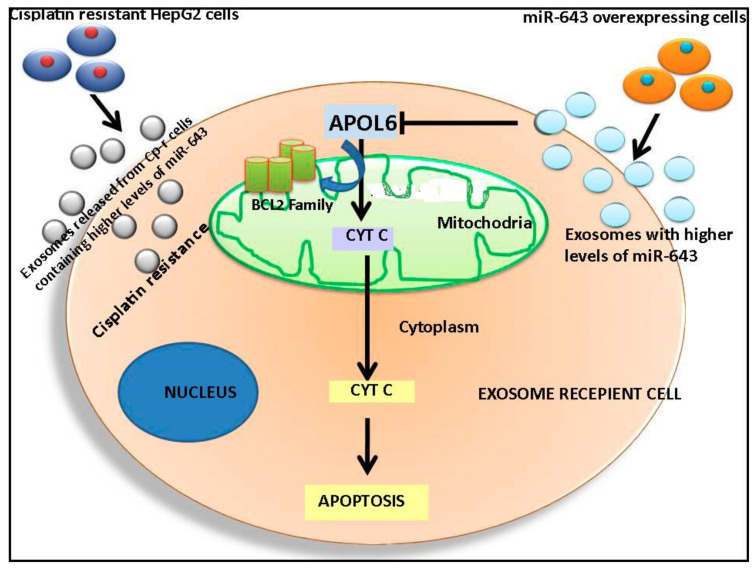
Schematic representation of mechanism of cisplatin resistance conferred by horizontal transfer of miR-643. Exosomes released from Cp-r HepG2 cells containing higher levels of miR-643 make the recipient Hela cells resistant to cisplatin. APOL6 is a major target of miR-643 and plays an important role in mitochondrial apoptosis [45]. Exosomes containing higher levels of miR-643 can target APOL6 and reverse APOL6 mediated apoptosis, thus promoting cell survival.

In conclusion, the data presented in this manuscript reveal that horizontal transfer of miR-643 confer resistance in Hela cells against cisplatin. Moreover, miR-643 conferred cisplatin resistance is, at least in part, mediated by the downregulation of APOL6, leading to inhibition of cellular apoptosis, thereby enhancing cell survival (Figure 12). The significance of miR-643 in mediating cisplatin resistance may have significant implications in designing combination therapy strategies involving anti-miR inclusions.

## Supplementary Materials

The following are available online at https://www.mdpi.com/article/10.3390/cells10061341/s1, Figure S1: Original full-length blots of APOL6 and loading control ACTIN from which Figure 5E(ii) was generated, Figure S2: Original full length blots of APOL6 and loading control ACTIN from which Figure 6B(II) was generated, Figure S3: Original full length blots of APOL6 and loading control ACTIN from which Figure 6C(II) was generated, Figure S4: Original full length blots of APOL6 and loading control ACTIN from which Figure 8A(II) was generated, Figure S5: Original Full length western blots of APOL6 and loading control ACTIN from which Figure 10A(I) was generated, Figure S6: Original full length blots of APOL6 and loading control ACTIN from which Figure 10B(I) was generated.
