# Supplementary material for: Horizontal Transfer of miR-643 from Cisplatin-Resistant Cells Confers Chemoresistance to Recipient Drug-Sensitive Cells by Targeting APOL6
Source: Cells. 2021 May 28;10(6):1341. doi: 10.3390/cells10061341 (PMC8229894; doi:10.3390/cells10061341)
Supplement: Supplementary file 1 [file cells-10-01341-s001.zip › cells-1183919-supplementary.pdf]

SUPPLEMENTARY FILE

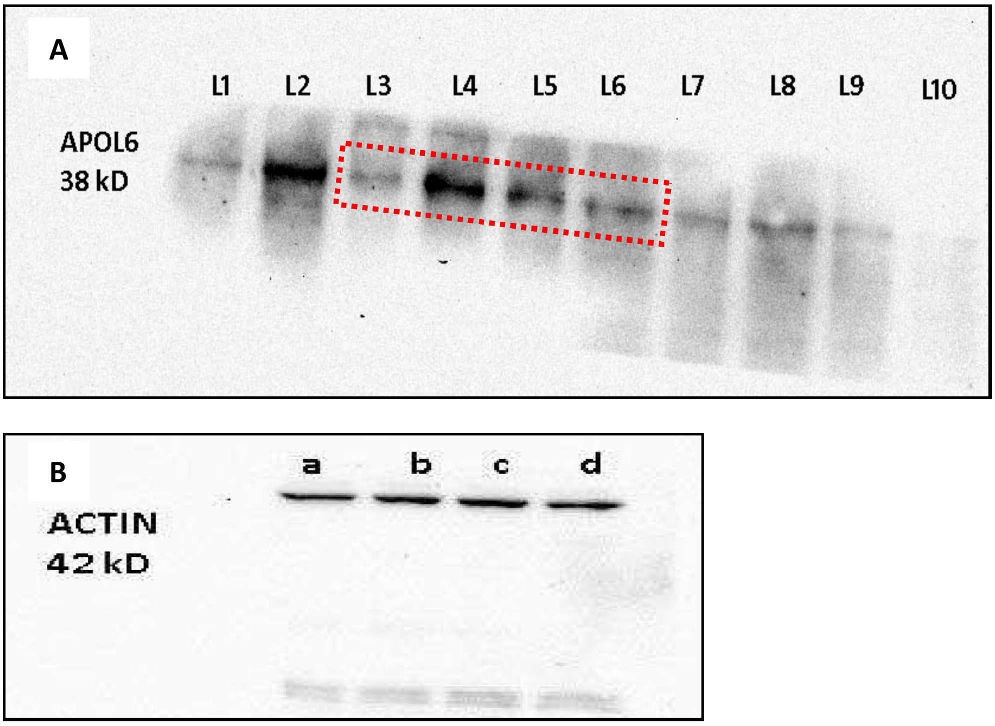

Figure S1. Original full-length blots of APOL6 and loading control ACTIN from which Figure 5 E(II) was generated. Hela cells were treated with EXsen or EXres in presence or absence of cisplatin followed by western blot analysis of the levels of APOL6 and ACTIN. **A.** Levels of APOL6 protein. In **A**, red dashed line box (L3, L4, L5 and L6) corresponds to a, b, c and d of Figure 5E(II) respectively. **B**. Levels of ACTIN protein. In A and B, a represents untreated cells; b represents cells treated with cisplatin; c represents cells treated with EXsen and cisplatin; d represents cells treated with EXres and cisplatin.

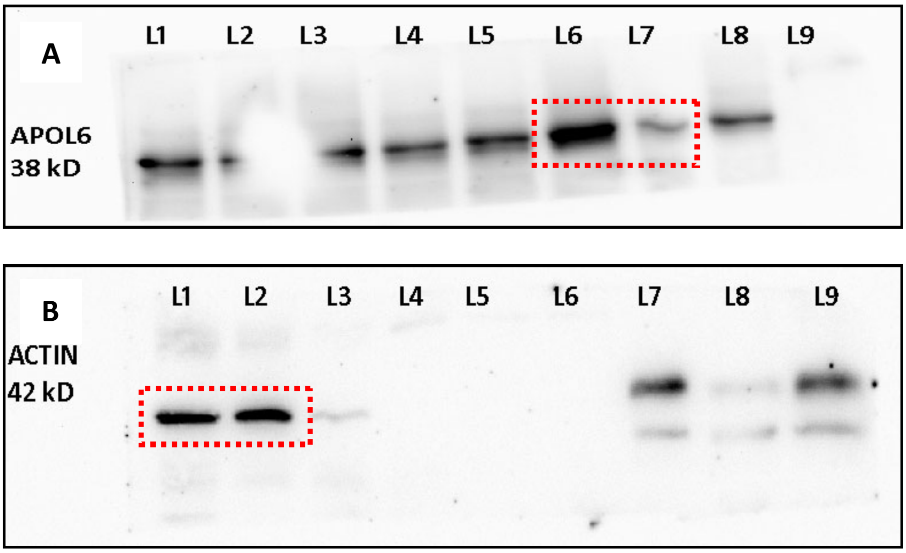

Figure S2. Original full length blots of APOL6 and loading control ACTIN from which Figure 6 B(II) was generated. Hela cells were transfected with pCMVmiR (control) or miR-643 construct in presence of cisplatin followed by western blot analysis of the levels of APOL6 and ACTIN. **A.** Levels of APOL6 protein. In **A**, red dashed line boxes (L6 and L7) corresponds to a and b of Figure 6 B(II) respectively **B**. Levels of ACTIN protein. In **B**, red dashed line box (L1 and L2) corresponds to a and b of Figure 6 B(II) respectively. In A and B, a represents pCMVmiR transfected cells treated with cisplatin; b represents miR-643 overexpressed cells treated with cisplatin.

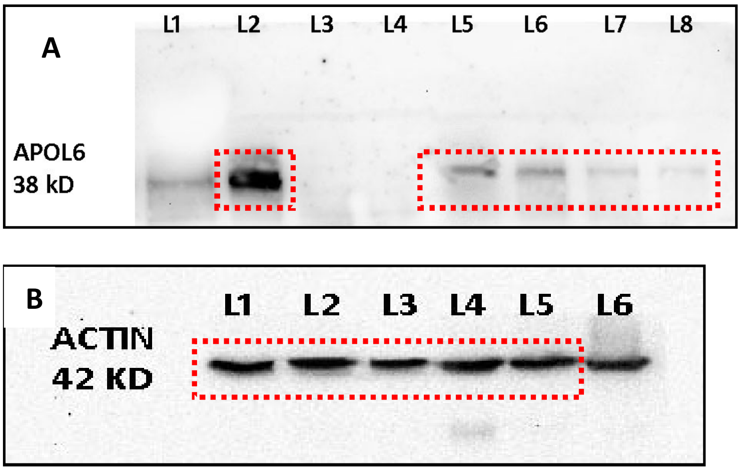

Figure S3. Original full length blots of APOL6 and loading control ACTIN from which Figure 6 C(II) was generated. miR-643 overexpressing Hela cells were treated with EXsen/EXres in presence of cisplatin followed by western blot analysis of the levels of APOL6 and ACTIN. **A.** Levels of APOL6 protein. In **A**, red dashed line box (L2, L5, L6, L7 and L8) corresponds to a, b, c, d and e of Figure 6 C(II) respectively. **B**. Levels of ACTIN protein. In **B**, red dashed line box (L1-L5) corresponds to a, b, c, d and e of Figure 6 C(II) respectively. In A and B, a represents pCMVmiR (vector) transfected cells treated with cisplatin; b represents pCMVmiR (vector) transfected cells treated with EXsen and cisplatin; c represents pCMVmiR (vector) transfected cells treated with EXres and cisplatin; d represents miR-643 overepressed cells treated with EXsen and cisplatin; e represents miR-643 overepressed cells treated with EXres and cisplatin.

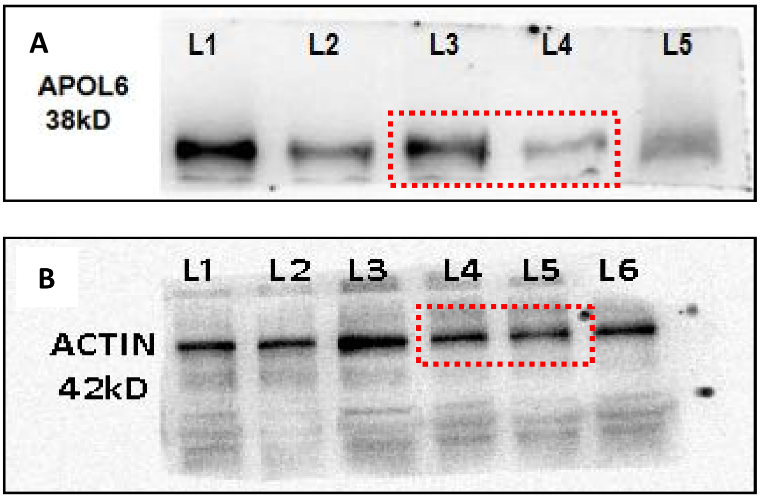

Figure S4. Original full length blots of APOL6 and loading control ACTIN from which Figure 8 A(II) was generated. miR-643 overexpressing Hela cells were treated with exosomes isolated from miR-643 overexpressing cells in presence of cisplatin followed by western blot analysis of the levels of APOL6 and ACTIN. **A.** Levels of APOL6 protein. In **A**, red dashed line box (L3 and L4) corresponds to a and b of Figure 8 A(II) respectively. **B**. Levels of ACTIN protein. In **B**, red dashed line box (L4 and L5) corresponds to a and b of Figure 8 A(II) respectively. In A and B, a represents Hela cells treated with $EXO_{pCMVmiR}$ + cisplatin; b represents Hela cells treated with $EXO_{miR-643}$ + cisplatin;

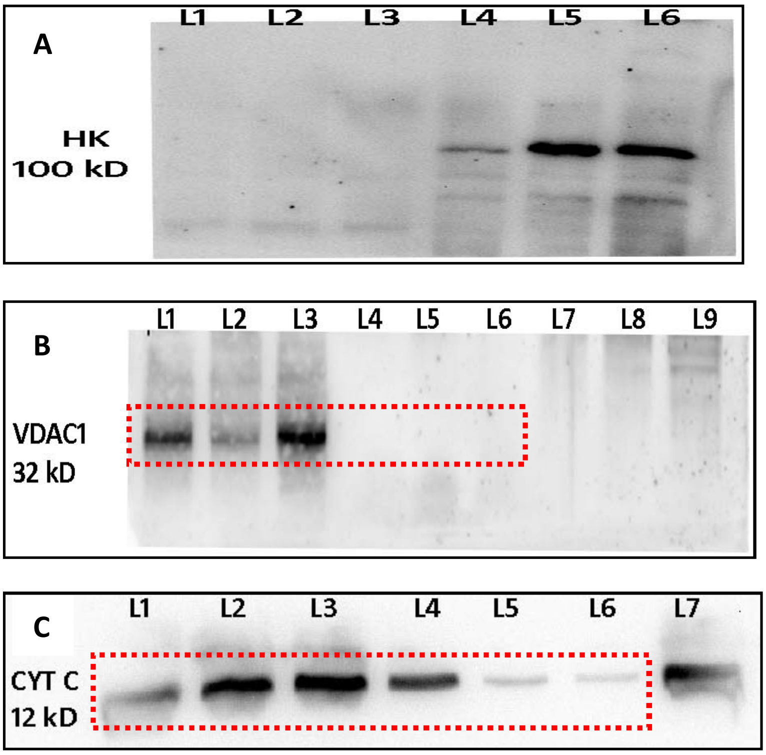

Figure S5. Original Full length western blots of APOL6 and loading control ACTIN from which Figure 10 A(I) was generated. Mitochondrial and cytoplasmic fractions extracted from Hela cells treated with EXsen/EXres and cisplatin followed by western blot analysis of the levels of HK, VDAC1 and CYT C. **A.** Levels of HK protein. In **A**, L1, L2, L3, L4, L5 and L6 corresponds to a, b, c, d, e and f of Figure 10 A(I) respectively. **B**. Levels of VDAC1 protein. In **B**, red dashed line box (L1-L6) corresponds to a, b, c, d, e and f of Figure 10 A(I) respectively. **C.** Levels of CYT C protein. In **C**, red dashed line box (L1-L6) corresponds to a, b, c, d, e and f of Figure 10 A(I) respectively. In A, B and C, a represents mitochondrial fractions of cells treated with cisplatin; b represents mitochondrial fractions of cells treated with EXsen and cisplatin; c represents mitochondrial fractions of cells treated with EXres and cisplatin; d represents cytoplasmic fractions of cells treated with cisplatin; e represents cytoplasmic fractions of cells treated with EXsen and cisplatin; f represents cytoplasmic fractions of cells treated with EXres and cisplatin;

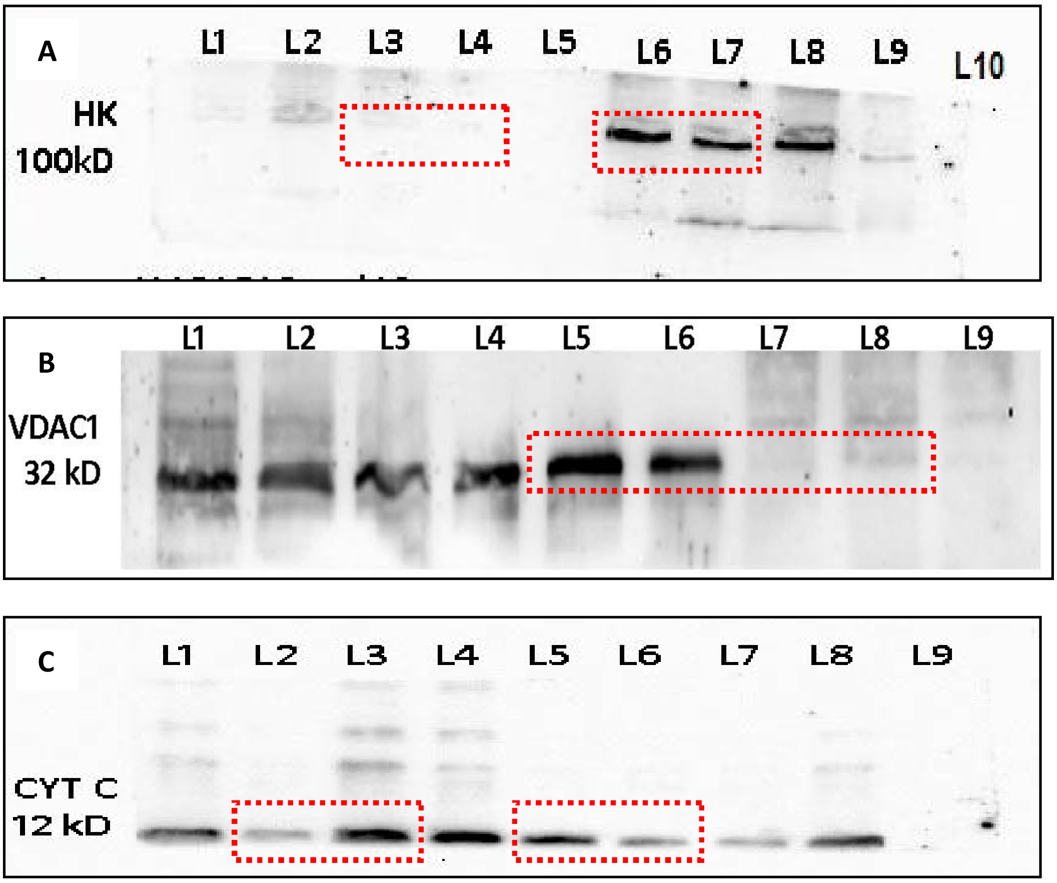

Figure S6. Original full length blots of APOL6 and loading control ACTIN from which Figure 10 B(I) was generated. Mitochondrial and cytoplasmic fractions extracted from miR-643 overexpressing Hela cells treated with cisplatin followed by western blot analysis of the levels of HK, VDAC1 and CYT C. **A.** Levels of HK protein. In **A**, red dashed line box (L3, L4, L6 and L7) corresponds to a, b, c and d of Figure 10 A(I) respectively. **B**. Levels of VDAC1 protein. In **B**, red dashed line box (L5-L8) corresponds to a, b, c and d of Figure 10 A(I) respectively. **C.** Levels of CYT C protein. In **C**, red dashed line box (L2, L3, L5, L6) corresponds to a, b, c and d of Figure 10 A(I) respectively. In A, B and C, a represents mitochondrial fractions of Hela cells treated with $EXO_{pCMVmiR}$ + cisplatin; b represents mitochondrial fractions of Hela cells treated with $EXO_{miR\text{-}643}$ + cisplatin; c represents cyoplasmic fractions of Hela cells treated with $EXO_{pCMVmiR}$ + cisplatin; d represents cytoplasmic fractions of Hela cells treated with $EXO_{miR\text{-}643}$ + cisplatin;
